# Determination of oxygenated and native polycyclic aromatic hydrocarbons in urban dust and diesel particulate matter standard reference materials using pressurized liquid extraction and LC–GC/MS

**DOI:** 10.1007/s00216-014-8304-8

**Published:** 2014-11-14

**Authors:** Trifa M. Ahmed, Christoffer Bergvall, Magnus Åberg, Roger Westerholm

**Affiliations:** Department of Analytical Chemistry, Arrhenius Laboratory, Stockholm University, 10691 Stockholm, Sweden

**Keywords:** OPAHs, PAHs, PLE, SRM 1649a, SRM 2975, SRM 1650b

## Abstract

The objective of this study was to develop a novel analytical chemistry method, comprised of a coupled high-performance liquid chromatography–gas chromatography/mass spectrometry system (LC–GC/MS) with low detection limits and high selectivity, for the identification and determination of oxygenated polycyclic aromatic hydrocarbons (OPAHs) and polycyclic aromatic hydrocarbons (PAHs) in urban air and diesel particulate matter. The linear range of the four OPAHs, which include 9,10-anthraquinone, 4*H*-cyclopenta[def]phenanthrene-4-one, benzanthrone, and 7,12-benz[a]anthraquinone, was 0.7 pg–43.3 ng with limits of detection (LODs) and limits of quantification (LOQs) on the order of 0.2–0.8 and 0.7–1.3 pg, respectively. The LODs in this study are generally lower than values reported in the literature, which can be explained by using large-volume injection. The recoveries of the OPAHs spiked onto glass fiber filters using two different pressurized liquid extraction (PLE) methods were in the ranges of 84–107 and 67–110 %, respectively. The analytical protocols were validated using the following National Institute of Standards and Technology standard reference materials: SRM 1649a (Urban Dust), SRM 1650b (Diesel Particulate Matter), and SRM 2975 (Diesel Particulate Matter, Industrial Forklift). The measured mass fractions of the OPAHs in the standard reference materials (SRMs) in this present study are higher than the values from the literature, except for benzanthrone in SRM 1649a (Urban Dust). In addition to the OPAHs, 44 PAHs could be detected and quantified from the same particulate extract used in this protocol. Using data from the literature and applying a two-sided *t* test at the 5 % level using Bonferroni correction, significant differences were found between the tested PLE methods for individual PAHs. However, the measured mass fractions of the PAHs were comparable, similar to, or higher than those previously reported in the literature.

**Electronic supplementary material:**

The online version of this article (doi:10.1007/s00216-014-8304-8) contains supplementary material, which is available to authorized users.

## Introduction

A vast body of scientific studies has shown associations between human exposure to urban air particulate matter (PM) pollution and severe health outcomes such as lung cancer, diseases of the respiratory and cardiovascular systems, premature mortality, and an increase in infant mortality [1–4]. The risks posed may be substantial; estimates from the Clean Air for Europe (CAFE) framework program indicate that approximately 350,000 premature deaths per year in the European Union can be attributed to exposure to PM [5]. A major source of PM in urban environments is motorized vehicles. The PM emitted from diesel-powered vehicles is especially considered to contribute to general health hazards [1, 3, 6–8], and in addition, diesel exhaust is classified as being carcinogenic to humans (Group 1) by the International Agency for Research on Cancer (IARC) [9]. However, the link between exposure to air PM pollution and adverse health effects has not yet been fully established, and consequently, it is important to characterize the chemical contents of air and diesel-derived PM [1, 3, 7]. Polycyclic aromatic hydrocarbons (PAHs) constitute a group of ubiquitous environmental pollutants present in air PM derived from different sources, and they are considered to play an important part in the adverse health outcomes due to PM exposure [6, 10]. Benzo[a]pyrene (B[a]P) is the only PAH currently classified as a human carcinogen, while many other PAHs are classified as possibly or probably carcinogenic (Groups 2A and 2B) to humans by the IARC [9]. In addition to cancer, PAHs have also been linked to reproductive and cardiovascular diseases [9, 10]. A group of less-studied PAH derivatives are oxygenated polycyclic aromatic hydrocarbons (OPAHs), which have recently been noted as being particularly reactive compounds with the potential of causing adverse biological effects. Due to their direct mutagenic potency, OPAHs may be more toxic to humans compared to their parent PAHs [11, 12]. OPAHs have higher molecular weights and lower vapor pressures than their parent PAHs [13], and thus, they have a higher tendency to be absorbed to PM in the atmosphere compared to their parent PAHs [14]. However, in a study on ambient air particles, it was shown that the low molecular weight OPAHs, naphthoquinone and anthraquinone, were more highly distributed in the gas phase [15]. In addition to their presence in the atmosphere, OPAHs have also been identified in PM from diesel and gasoline exhaust [16, 17]. PM from the atmosphere and from vehicle exhaust is a complex chemical matrix with OPAHs present at trace levels. Consequently, for methods aimed at the determination of OPAHs, there are high demands on both the selectivity and detection limits. Various different approaches have been used in analyzing OPAHs, which have been recently reviewed by Walgraeve and co-workers [18]. Typically, the OPAHs associated with PM are solvent extracted using various techniques such as Soxhlet extraction [19], ultrasonically assisted extraction [20, 21], supercritical fluid extraction [22], and pressurized liquid extraction (PLE) [23–25]. PLE has been shown to give an equivalent or higher extraction efficiency compared to Soxhlet extraction for PAHs from air and diesel PM standard reference materials (SRMs) and recently for OPAHs from a diesel PM SRM [26].

Chemically complex extracts of PM usually require one or several cleanup steps prior to the final analysis. Generally, sample cleanup is labor intensive and time consuming, and advantages could be gained by the online coupling of the sample pre-treatment and the analysis. Increasing the degree of automation in analytical protocols reduces manual sample handling and reduces the risk of errors due to cross contamination and analyte losses.

An alternative to manual cleanup and subsequent gas chromatography (GC) analysis is the online coupling of HPLC and GC, which has been extensively reviewed by Hyötyläinen and Riekkola [27]. Online HPLC–GC combines the flexible and selective cleanup capabilities of HPLC with the high peak resolution of GC. By using large-volume injection (LVI), a major part of the sample can be injected, thereby lowering the detection limits in comparison with traditional GC injection techniques where only a small part of the sample (~1 μl) is introduced into the GC. Furthermore, the HPLC separation can be easily monitored using a UV detector allowing for precise fractions to be transferred to the GC.

The coupling of liquid chromatography (LC) to GC/mass spectrometry (MS) for partial sample cleanup, separation, and detection has been successfully applied for the analysis of PAHs in environmental matrices [28, 29].

The aim of the present study was to evaluate and optimize a methodology comprised of PLE, initial sample cleanup using solid-phase extraction (SPE), and automated cleanup, separation, and detection using LC–GC/MS for the determination of the OPAHs in air and diesel PM.

## Experimental

### Chemicals and solvents

The solvents used in this present study were toluene, methanol, hexane, methyl *tert*-butyl ether (HPLC grade, Rathburn Chemicals Ltd, UK), ethanol (absolute, Ph. Eur., VWR International S.A.S, Fontenay-sous-Bois, France), and anhydrous dodecane (>99 %, Sigma-Aldrich, St. Louis, MO, USA).

The OPAH calibration standard solutions used for quantification and identification consisted of perdeuterated anthraquinone-D_8_ (AQ-D_8_) 99.4 % as an internal standard, 9,10-anthraquinone (ANQ) >99.5 %, 4*H*-cyclopenta[def]phenanthren-4-one (CCPQ) >99 %, benzanthrone (BAQ) >98.5 %, and 7,12-benz[a]anthraquinone (BaAQ) 97.5 % dissolved in toluene, and they were all purchased as solutions from Chiron AS (Trondheim, Norway). Hexane was initially used as the diluent, but it was observed that the OPAHs precipitated, and therefore, toluene was selected for the dilutions. Perylene (see Electronic Supplementary Material (ESM) Table S1) was used as a volumetric internal standard for the recovery experiments on SPE, and Cor-D_12_ was added as a volumetric internal standard to the second extracts from each sample for quantitative analyses of the PLE extracts. AQ-D_8_ was used as a volumetric internal standard for the determination of the recoveries for the spiked PLE filter experiments. A list of the PAHs and internal standards used in this study along with their abbreviations and suppliers is given in ESM Table S1 in the supporting information. All of the standard solutions were stored in a freezer at −18 °C prior to use. Before use, the standard solutions were ultrasonicated at room temperature for 10–15 min. Furthermore, laboratory UV filters (LF 101 yellow, Bellialite, Sweden) were used for covering both the windows and light sources in the laboratory to eliminate the potential breakdown of the analytes [30].

### Standard reference materials

The standard reference materials used in this present study were the following: SRM 1649a (Urban Dust) [31], SRM 1650b (Diesel Particulate Matter) [32], and SRM 2975 (Diesel Particulate Matter, Industrial Forklift) [33], all of which were purchased from the National Institute of Standards and Technology (NIST, Gaithersburg, MD, USA).

### Pressurized liquid extraction of OPAHs and PAHs

Three replicate samples of each individual SRM, which corresponded to 10 mg of SRM 1649a, 20 mg of SRM 1650b, and 20 mg of SRM 2975, were weighed on glass microfiber filters (GF/C, *Ø* = 47 mm, Whatman International Ltd, England) using an analytical balance with a precision of ±0.001 mg. The filters were inserted into the extraction cells, and the following perdeuterated internal standards were added: AQ-D_8_, Phe-D_10_, Pye-D_10_, B[a]A-D_12_, B[a]P-D_12_, B[ghi]P-D_12_, and DB[ai]P-D_14_ (see ESM Table 1S for the abbreviations).

The PM samples were extracted using an accelerated solvent extraction system (ASE 200, Dionex Corporation, Sunnyvale, CA, USA). Three different extraction procedures, i.e., E1, E2, and E3, were employed, and the extraction parameters used are shown in Table 1. To investigate the efficiency of the extraction procedure, each sample was extracted an additional time using the same extraction parameters used in the first extraction.

**Table 1:** Pressurized liquid extraction parameters used in this study

Extraction method	Temperature (°C)	Pressure (MPa)	Preheat time (min)	Static cycles analytical extraction	Static time (min)	Flush volume (%)	Extraction solvent
E1	200	20.6	3	5	30	30	Tol:MeOH (9:1)
E2	200	13.8	No	3	5	30	Tol
E3	200	13.8	No	3	30	30	Tol

*Tol* toluene, *MeOH* methanol

### Isolation of OPAHs and PAHs

The PLE extracts were concentrated to approximately 5 ml under a gentle gas stream of nitrogen gas while being heated to 60 °C in a water bath (TurboVap® LV evaporator, Zymark, Hopkinton, MA, USA). The concentrated extracts were then transferred to disposable test tubes and evaporated further to volumes of approximately 0.5 ml. Silica SPE cartridges (100 mg Isolute, IST, UK) were conditioned with 3 ml of hexane prior to use. The concentrated extracts were then added onto the conditioned SPE cartridges, and the test tubes were rinsed with an additional 0.5 ml aliquot of toluene, which was also added onto the SPE cartridge. Subsequently, the analytes were eluted with 1.5 ml of toluene into a disposable test tube. This fraction was then evaporated to approximately 0.5 ml using a very gentle nitrogen stream, and it was then divided into two aliquots, which were subsequently transferred into two 300 μl microvials. One vial was directly used for OPAH analysis, while the second extract was further reduced to approximately 0.1 ml, diluted with hexane to approximately 0.3 ml, and analyzed for PAHs.

### LC–GC/MS analysis

The hyphenated online LC–GC/MS system used for the separation and detection of OPAHs and PAHs consisted of a CMA/200 microsampler (CMA Microdialysis AB, Sweden), an HPLC pump (Varian Inc, Palo Alto, CA, USA), a UV detector (SPD-6A, Shimadzu, Japan), and a normal phase LC column (Nucleosil 100-5NO_2_ 124 × 4.6 mm, 5 μm). The GC/MS system consisted of an Agilent 6890N gas chromatograph (Agilent Technologies, Palo Alto, CA, USA) with an Agilent 5973N MSD (Agilent Technologies). The LC column was used as a cleanup step for removing alkanes and monoaromatic compounds using the back flush technique. The cleaned-up PAH and OPAH fraction was transferred online through a transfer line to the programed temperature vaporizer (PTV) GC injector. The PTV was operated in the solvent vent mode in order to evaporate the LC effluent. This system allows for automated sample cleanup, and LVI has previously been used for PAH analysis; a description of the system setup and operation can be found outlined in detail elsewhere [28, 29]. Operation of the LC–GC/MS system for PAH analysis was performed in accordance with the parameters outlined by Sadiktsis et al. [34]. Furthermore, for the PAHs determined in this study, details on the calibration data, such as the linear range and coefficients of determination from the calibration curves, LODs, and LOQs, have recently been reported in detail [34].

**Fig. 1:**
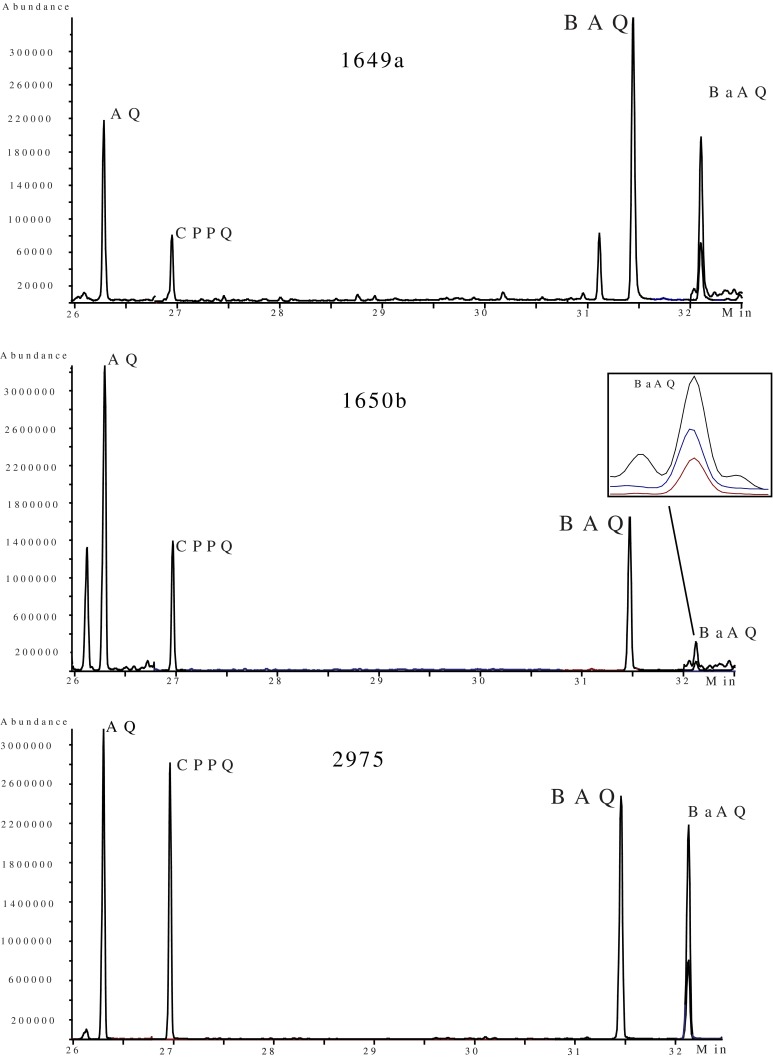
GC/MS chromatograms (SIM) for the OPAHs in SRM 1649a. SRM 1650b (extracted ions for BaAQ are 258, 230, 222) and SRM 2975

The same LC–GC/MS method was used for the analysis of OPAHs except that the LC mobile phase was changed to a mixture of hexane and methyl *tert*-butyl ether (8:2, *v*/*v*) with 0.1 % dodecane added, and the time for reversing the flow through the LC column was adjusted depending on the retention time for AQ-D_8_. The large injection volume is a back flush peak from the LC column monitored using the UV detector to transfer most of the part of interest onto the GC/MS (≥95 % for PAHs and ≥91 % for OPAHs). The MS system was set to acquire data in the selected ion monitoring (SIM) mode. The molecular ion and two analyte-specific fragments were monitored for each OPAH (see Table 2). The peak identities of the OPAHs in the SRM samples were confirmed by the retention times and relative ion ratios compared to the standard solutions. Concentrations of the PAHs and OPAHs in the SRM samples were calculated using the peak areas of the most abundant ions (see Fig. 1) and the relative response factors established from the calibration curves. Glass microfiber filters served as method blanks for the OPAHs and PAHs and were treated in the same manner as the samples throughout the entire analytical procedure.

**Table 2:** Silica SPE recoveries (mean percentage) of four replicate experiments, coefficient of determination for the linear calibration curves using seven-point calibrations on the LC–GC/MS system, and ions selected for the SIM method

Quinone standards	% Mean (STD)	*R* ^2^	*m*/*z*		
			Molecular ion	First fragment	Second fragment
AQ-D_8_	83.4 (3.2)	0.995	216	188	160
AQ	89.3 (3.7)	0.995	208	180	152
4HCPPQ	107.3 (5.2)	0.996	204	176	150
BAQ	87.0 (4.8)	0.992	230	202	174
BaAQ	87.4 (6.2)	0.987	258	230	202

*STD* standard deviation

### Statistical significance

The measured mass fractions were compared for statistically significant differences using two-sided *t* tests at the 5 % level with Bonferroni correction. The Bonferroni correction used *n* = number of analytes for which the method comparison was made. Typically, this number ranged from 4 to 44 depending on the SRM and whether the targeted analytes were OPAHs or PAHs. The methods were also compared for systematical differences, e.g., that the concentrations of all of the analytes are consistently lower using one extraction method compared to another, by testing if the distribution showed deviates from what can be expected from a binomial distribution with equal probabilities for the two outcomes.

## Results and discussion

### Sample preparation

Three different PLE procedures were used in this study for extracting the OPAHs and PAHs from the SRMs, and details on the PLE methods are presented in Table 1. Extraction procedure E1 is an exhaustive extraction protocol previously developed by our research group [35], and it has previously been used for the determination of PAHs in diesel SRMs [34, 36], whereas extraction procedures E2 and E3 are adopted from a recent paper from NIST using PLE for the extraction of PAHs and nitro PAHs from air and diesel PM SRMs [36]. Recoveries using the two most exhaustive extraction methods, E1 and E3, were calculated by spiking glass microfiber filters with a standard mixture of the OPAHs (Table 3). The results show that the two extraction methods recover between 67 and 110 % of the four OPAHs, as is shown in Table 3. Higher recoveries and lower standard deviations were obtained with the less-exhaustive method E3 compared to method E1 (Table 3). These results correspond with recent studies using PLE [24, 37]. Mirivel and co-workers determined the PLE recoveries of various OPAHs (including BAQ and BaAQ) spiked onto filters to be in the order of 79–107 % (PLE with methanol and one 8-min static cycle at 100 °C) [24], and Walgraeve and co-workers obtained recoveries of 99 ± 4 % and 101 ± 4 % for BAQ at ambient temperature and at 40 °C, respectively (PLE with ethyl acetate and three 5-min static cycles). The corresponding recoveries for BaAQ were reported to be 110 ± 14 % and 95 ± 3 % at ambient air temperature and 40 °C, respectively. The somewhat lower extraction recovery obtained for BaAQ compared to the data presented by Walgraeve and co-workers could be a result of the higher extraction temperature and longer extraction time used in the present study. Although no significant difference in the extraction recovery of BaAQ was obtained by Walgraeve and co-workers at ambient temperature compared with that at 40 °C, the authors suggest that the lower extraction recoveries and higher standard deviations obtained for other OPAHs such as benzo[a]pyrene-4,5-dione, chrysene-5,6-dione, and phenanthrene-9,10-dione at 40 °C compared with those at ambient temperature could be a result of thermal degradation [37]. Additionally, Lintelmann and co-workers found low extraction recoveries (<80 %) of OPAHs spiked onto filters when using PLE with toluene, methanol, or hexane/acetone at temperatures ≤100 °C, which the authors attributed to degradation or chemical reaction processes [38]. However, none of the OPAHs used in our present study were investigated. Consequently, the lower extraction recoveries and higher standard deviations found in this study for BaAQ when using extraction procedure E1, as shown in Table 3, could be due to having 10 % methanol and/or E1 being more exhaustive than E3 (the use of five extraction cycles in E1 compared to the use of three extraction cycles in E3).

**Table 3:** PLE recoveries (mean percentage of three replicate experiments) of standards spiked to glass microfiber filters

Quinone standards	E1	E3
	% Mean (STD)	% Mean (STD)
AQ	97.0 (12.9)	105.8 (0.6)
4HCPPQ	110.0 (14.6)	105.8 (4.7)
BAQ	93.1 (15.4)	107.6 (10.0)
BaAQ	67.2 (17.5)	84.0 (8.9)

*STD* standard deviation

Initially, hexane was used as the solvent for eluting the OPAHs from the SPE cartridges using a methodology previously reported for PAHs [27, 28]. However, applying this scheme for the OPAHs resulted in poor analyte recoveries, and subsequently, toluene was tested and selected as a solvent. An elution volume of 2 ml of toluene was established as satisfactory by incremental additions of 0.5 ml toluene. Using this SPE method gave recoveries above 83 % for the OPAHs in this study, as shown in Table 2.

### Method calibration

The LC–GC/MS system was calibrated using seven-point calibration curves with three replicate injections at each point for all of the OPAHs. The GC/MS peak areas for the five OPAHs increased linearly with the injected amount over the range of 0.7 pg to 43.3 ng, displaying an *R* ^2^ ≥ 0.986 as shown in Table 2.

The instrumental limits of detection (LOD) were established by the stepwise dilution of the standard solutions used as the last points in the calibration curves until peaks having signal to noise ratios (*S*/*N*) of ≥3 were achieved, and the limits of quantification (LOQ) were the last points in the calibration curves with *S*/*N* of ≥10. Both the LODs and LOQs were calculated by the Agilent ChemStation data analysis software (E.02.02.1431) and are shown in Table 4 together with the reported literature data on the LODs for the analytes.

**Table 4:** Instrumental LOD and LOQ in picograms obtained in the present study compared with LOD values from the literature

OPAHs	LOD, this work	LOQ, this work	LOD, Walgraeve et al. [37]	LOD, Layshock et al. [25]	LOD, Cho et al. [44]	LOD, Mirivel et al. [24]	LOD, Delhomme et al. [21]	O’Connell et al. [39]^a^
AQ-d8	0.06	0.13	ND	ND	ND	ND	ND	0.65
AQ	0.6	1.2	ND	5	1500	10	18.4	6.9
4HCPPQ	0.2	0.7	ND	1	ND	ND	ND	0.21
BAQ	0.6	0.9	8	10	ND	53	3.4	0.78
BaAQ	0.8	1.3	926	10	ND	215	4	0.85

*ND* no data reported

^a^GC/MS

The LODs for the OPAHs in our work are similar to, equal to, or substantially lower than values reported in previous studies using GC/MS, LC/MS, and LC/MS/MS methodologies (Table 4). The LOD for AQ-D_8_ is ten times lower than the LOD for AQ in this present study, and a similar difference in the LODs for these OPAHs can be seen in O’Connell et al. [39]. Albinet and co-workers have reported LODs for OPAHs (including AQ) in the range of 0.01–2.60 pg [23]. The low LODs obtained in their study are most likely a result of operating the MS in the negative ion chemical ionization mode.

### Method validation

#### OPAH levels in SRMs using different PLE conditions

Different PLE methods (E1, E2, and E3; see Table 1) were used for the extraction of the OPAHs from SRMs 1649a, 1650b, and 2975. SRM 1649a was extracted using extraction protocols E1 and E2 whereas the diesel PM SRMs were extracted using extraction methods E1 and E3. The rationale for this experimental setup was that it has been previously shown that PAHs are more difficult to extract from diesel PM compared to air PM and require high-temperature PLE with long extraction times [35]. In addition, Nocun and Schantz concluded that PLE seems to be a more effective extraction method for the extraction of OPAHs from diesel PM compared to Soxhlet extraction [26]. Our results show that higher values were obtained for all of the OPAHs studied in SRM 1649a using the more-exhaustive extraction method E1 (with 10 % methanol) compared to the data generated by extraction method E2 (Table 5). However, the increase was only significant for AQ using Student’s *t* test at the 5 % probability level (*p* = 0.02, two-sided test, Bonferroni corrected with *n* = 4 for the number of analytes tested) as shown in Table 6.

**Table 5:** Mass fractions in nanograms per milligram of SRM 1649a (Urban Dust) determined in the present work (*n* = 3, with standard deviations) compared to data from previous studies

OPAHs	This work, E1	This work, E2	Fernandez and Bayona [45]	Mirivel et al. [24]	Albinet et al. [23]	Durant et al. [46]	Cho et al. [44]	Sun et al. [47]	Nocun and Schantz [26]
AQ	3.67 (0.23)	3.23 (0.027)	2.2 (0.04)	2.357 (0.183)	2.238 (0.363)	2.70 (0.12)	2.03(0.192)	2.051	1.39 (0.16)
4HCPPQ	0.949 (0.138)	0.867 (0.039)	0.17 (0.05)	ND	ND	0.47 (0.06)	ND	ND	1.14 (0.09)
BAQ	5.71 (0.25)	4.47 (0.52)	1.310 (0.02)	4.66 (0.460)	3.715 (0.872)	4.50 (0.34)	ND	2.145 (0.128)	3.13 (0.40)
BaAQ	5.51 (0.40)	5.20 (0.80)	7.465 (1.1)	3.44 (0.322)	8.459 (0.797)	2.40 (0.25)	ND	5.588 (1)	3.75 (0.19)

*ND* no data reported

**Table 6:** Comparison of the determined OPAHs from the present study by two-sided *t* tests with Bonferroni correction. The table lists significant differences for comparisons of extraction methods. Single (+/−) and double (++/−−) indicate statistically significant differences on the 5 % level without and with Bonferroni correction, respectively. The single plus or minus differences can be seen as an indication of a potential difference, while double plus or minus indicate stronger evidence for a true difference. No symbol indicates that no differences could found

OPAHs	1649a	1650b	2975
	E1/E2	E1/E3	E1/E3
AQ	++		++
4HCPPQ			−−
BAQ			++
BaAQ		++	

For the diesel PM SRM 1650b, extraction method E3 gave higher mass fractions for AQ and 4CPPQ, while extraction method E1 gave higher mass fractions for BAQ and BaAQ (Table 7). For SRM 2975, higher concentrations were observed using E1 compared to the less-exhaustive E3 method except for 4CPPQ (Table 8). The observed differences between E1 and E3 were significant for BaAQ in SRM 1650b (mass fraction ratio 1.0, *p* = 0.002) and for all of the OPAHs but BaAQ in SRM 2975 (mass fraction ratios 0.90–1.3, *p* = 0.0005–0.01). Significance was assessed by two-sided *t* tests with Bonferroni correction (*n* = 4), and the results are shown in Table 6.

**Table 7:** Mass fractions in nanograms per milligram of SRM 1650b (Diesel Particulate Matter) determined in the present work (*n* = 3, with standard deviations) compared to data from previous studies

OPAHs	This work, E1	This work, E3	NIST reference value [43]	Mirivel et al. [24]	Layshock et al. [25]	Nocun and Schantz [26]	O’Connell et al. [39]^a^	O’Connell et al. [39]^b^
AQ	58.5 (0.6)	60.3 (0.7)	ND	37.54 (3.941)	47.7 (19.9)	53.11 (1.70)	64	21
4HCPPQ	14.4 (0.9)	17.2 (1.2)	15.6 (0.6)	ND	6.90 (4.1)	16.88 (0.90)	9.2	7.6
BAQ	27.1 (2.3)	19.6 (3.5)	ND	8.73 (1.016)	36.90 (8.6)	16.03 (0.34)	23	13
BaAQ	8.74 (0.68)	4.94 (0.45)	ND	<3.996	BDL	8.90 (1.02)	5.7	5.1

*ND* no data reported, *BDL* = below the detection limit

^a^GC/MS

^b^LC/MS

**Table 8:** Mass fractions in nanograms per milligram of SRM 2975 (Diesel Particulate Matter) determined in the present work (*n* = 3, with standard deviations) compared to data from previous studies

OPAHs	This work, E1	This work, E3	Cochran et al. [40]	Nocun and Schantz [26]
AQ	39.1 (0.3)	36.7 (0.2)	8.710 (0.33)	15.95 (2.73)
4HCPPQ	20.9 (0.3)	24.0 (0.4)	ND	19.95 (2.51)
BAQ	28.5 (0.4)	22.2 (1.7)	ND	16.28 (1.22)
BaAQ	41.4 (1.5)	38.0 (1.9)	ND	21.93 (1.96)

*ND* no data reported

The higher mass fractions obtained using E1 compared to E2 or E3 are likely to be due to the more-exhaustive extraction conditions and/or the use of 10 % methanol in the toluene in E1. The somewhat lower concentrations obtained for 4HCCPQ in the diesel PM SRMs when using extraction method E1 could be a result of degradation.

Interestingly, the obtained PLE recoveries for spiked filters do not reflect the concentrations determined in the SRMs. For example, although not statistically significant (high standard deviations), a higher recovery was obtained for BaAQ when using E3 compared to when using E1, while the opposite was found for SRM 1650b, which yielded a much higher mass fraction when using E1 compared to E3. These types of differences have previously been reported by Lintelmann [20]. The author assumed that the matrix can have a role in deactivation of the active site on the quartz filter, which may stabilize the OPAHs.

#### Comparison of OPAH concentrations with the literature data

Data on previously published OPAH concentrations are given in Tables 5, 7, and 8 together with the values generated in the present study. Generally, the concentrations obtained in our study are similar to or higher than the data reported from previous studies. Unfortunately, no certified values have been assigned for these compounds by the NIST in the SRMs tested. A reference value of 15.6 ± 0.6 ng/mg exists for 4HCCPQ in SRM 1650b (Table 7), which correlates well with the concentrations generated in the present study, i.e., 14.4 ± 0.9 ng/mg when extracted using E1 and 17.2 ± 1.2 ng/mg when using E3, respectively. In reviewing the literature data from different studies, there are large variations in the reported mass fractions of the four OPAHs (Table 5). The differences could arise from all of the steps of the analytical protocols employed. In a recent study by O’Connell and co-workers [40], they compared the results obtained from analyzing SRM extracts using both GC/MS and LC/MS, and they concluded that the GC–MS method is preferred over the LC–MS method for quantification of the OPAHs.

Apart from using GC/MS or LC/MS, the differences in the mass fractions could arise from the choice of the extraction method (as discussed above) or which internal standard was used for calibration. Only a few isotope-labeled OPAHs are commercially available at the moment, and in some previous studies, isotope-labeled PAHs or nitro PAHs have been used as internal standards [41].

Nocun and Schantz reported differences in the mass fraction obtained for AQ when analyzing air PM SRM extracts using two different GC columns, one 50 % phenyl methylpolysiloxane column (DB-17MS) and one low-polarity column (DB-XLB) [26]. They suggested that the higher values reported in previous studies for AQ and BAQ could be caused by co-elution with other isomers due to the shorter columns that were used with a low-polarity stationary phase and/or faster temperature ramps. The authors concluded that the DB17-MS capillary column is the better choice as it gives less co-elution.

In the present study, a 60-m DB17-MS capillary column was used, and the peak identification of the samples was performed by comparing the area ratios of three fragment ions for each OPAH with those obtained from the standards to reduce the risk of the overestimation of the mass fractions as a result of interfering compounds. The higher amounts of OPAHs found in our study compared to the results presented by Nocun and Schantz are likely a result of other factors such as the extraction method as well as using SPE as a cleanup step.

#### PAHs

Aliquots of the same SPE extracts used for the analyses of the OPAHs were injected onto the LC–GC/MS system for quantitative PAH analysis. The results for the PAHs listed as priority PAHs by the EU [41] and the US EPA [42] are presented in Tables 9, 10, and 11 together with the NIST reference/certified values and other data taken from the literature. The mass fractions determined for additional PAHs in the SRM samples are shown in the ESM in Tables S2 to S4. A statistical evaluation of PAH concentrations using the E1, E2, and E3 PLE protocols as well as with NIST and literature data were performed with the two-sided Student’s *t* test. The results are shown in Table 12 for the selected EU/EPA PAHs and in ESM Table 5S for the additional PAHs analyzed.

**Table 9:** Mass fractions in nanograms per milligram for SRM 1649a (*n* = 3, with standard deviations) using E1 and E2 methods, certified/reference values for SRM 1649a assigned by NIST and data for SRM 1649b by Schantz

PAHs	Abbreviations	This work, E1	This work, E2	NIST [31]	Schantz et al. [26]^c^
Phenanthrene	Phe	5.36 (0.50)	5.12 (0.35)	4.14 (0.37)^a^	4.354 (0.041)
Anthracene	Ant	0.809 (0.086)	0.841 (0.156)	0.432 (0.082)^a^	0.965 (0.008)
Fluoranthene	Flu	6.72 (0.69)	6.52 (0.18)	6.45 (0.18)^a^	6.571 (0.079)
Pyrene	Pyr	5.55 (0.43)	5.33 (0.25)	5.29 (0.25)^a^	4.969 (0.048)
Benz[a]anthracene	B[a]A	2.58 (0.23)	2.48 (0.17)	2.208 (0.073)^a^	2.268 (0.020)
Chrysene	Chr	3.63 (0.13)	3.59 (0.08)	3.049 (0.06)^a^	2.988 (0.038)
Benzo[b]fluoranthene	B[b]F	6.37 (0.14)	6.11 (0.15)	6.45 (0.64)^a^	7.760 (0.220)^d^
Benzo[k]fluoranthene	B[k]F	1.82 (0.17)	2.14 (0.07)	1.913 (0.031)^a^	1.870 (0.10)
Benzo[a]pyrene	B[a]P	2.63 (0.20)	2.49 (0.18)	2.509 (0.087)^a^	2.970 (0.110)
Indeno[1,2,3-cd]pyrene	I[1,2,3-cd]P	2.40 (0.01)	2.62 (0.07)	3.180 (0.72)^a^	2.678 (0.028)
Dibenz[a,h]anthracene	DB[a,h]A	0.534 (0.052)	0.326 (0.120)	0.288 (0.023)^a^	0.573 (0.013)^e^
Dibenzo[a,l]pyrene	DB[al]P	0.035 (0.019)	0.053 (0.022)	0.612 (0.0074)^b^	ND
Dibenzo[a,e]pyrene	DB[ae]P	0.429 (0.159)	0.513 (0.033)	0.565 (0.060)^a^	0.622 (0.042)
Dibenzo[a,i]pyrene	DB[ai]P	0.135 (0.035)	0.180 (0.034)	ND	ND
Dibenzo[a,h]pyrene	DB[ah]P	0.053 (0.012)	0.058 (0.007)	0.047 (0.010)^a^	ND

*ND* no data reported

^a^Certified mass fraction for PAHs in SRM 1649a

^b^Reference mass concentrations for PAHs in SRM 1649a

^c^Method 8 (parameters in this PLE method are the same as those of E2)

^d^Sum of B[b]F and B[j]F

^e^Sum of DB[a,c]A and DB[a,h]A

**Table 10:** Mass fractions in nanograms per milligram for SRM 1650b (*n* = 3, with standard deviations) for the PAHs extracted from 1650b using E1 and E3 methods and certified/reference values assigned by NIST and data from the literature

PAHs	This work, E1	This work, E3	NIST [32]	Schantz et al. [36]^c^	Schantz et al. [36]^d^	Sadiktsis et al. [34]^e^
Phe	65.9 (2.3)	79.2 (1.4)	69.5 (1.9)^a^	71.090 (0.045)	71.680 (1.260)	72.7 (1.4)
Ant	6.12 (0.96)	12.2 (0.4)	7.67 (0.47)^a^	7.47 (0.13)	7.450 (0.310)	6.79 (0.32)
Flu	42.0 (0.8)	45.6 (1.2)	47.3 (0.8)^a^	50.2 (0.50)	50.780 (940)	44.0 (0.6)
Pyr	35.5 (1.0)	40.9 (1.9)	43.4 (1.6)^a^	45.90 (0.66)	45.120 (2.560)	38.1 (0.4)
B[a]A	7.40 (0.28)	7.17 (0.11)	6.18 (0.30)^a^	7.87 (0.27)	7.960 (0.110)	6.66 (0.08)
Chr	15.2 (0.2)	16.2 (0.6)	13.3 (1.1)^a^	13.76 (0.37)	13.130 (0.680)	15.9 (0.4)
B[b]F	8.88 (0.65)	7.54 (0.42)	6.77 (0.84)^a^	ND	ND	8.43 (0.4)
B[k]F	2.69 (0.17)	2.25 (0.10)	2.37 (0.21)^a^	2.22 (0.07)	2.230 (0.046)	2.75 (0.07)
B[a]P	1.56 (0.09)	1.38 (0.10)	1.17 (0.09)^a^	1.68 (0.15)	1.680 (0.060)	1.40 (0.03)
I[1,2,3-cd]P	4.15 (0.36)	3.66 (0.39)	4.44 (0.28)^a^	4.59 (0.07)	4.400 (0.230)	3.49 (0.23)
DB[a,h]A	0.436 (0.068)	0.346 (0.061)	0.365 (0.071)^a^	ND	ND	0.586 (0.078)
DB[al]P	0.061 (0.022)	0.037 (0.015)	0.137 (0.024)^b^	ND	ND	0.0178 (0.0030)
DB[ae]P	0.569 (0.071)	0.599 (0.059)	1.14 (0.12)^b^	1.18 (0.08)	1.130 (0.060)	0.607 (0.043)
DB[ai]P	0.049 (0.014)	0.056 (0.007)	ND	ND	ND	0.0470 (0.0019)
DB[ah]P	0.022 (0.004)	0.030 (0.003)	ND	ND	ND	0.0229 (0.0070)

*ND* no data reported

^a^Certified mass fraction for PAHs in SRM 1650b

^b^Reference concentrations for PAHs in SRM 1650b

^c^Method 12 (parameters in this PLE method are the same as those of E3)

^d^Method 17 (parameters in this PLE method are the same as those of E1)

^e^PLE parameter the same as that of E1

**Table 11:** Mass fractions in nanograms per milligram for SRM 2975 (*n* = 3, with standard deviations) for the PAHs extracted from 2975 with ASE using E1 and E3 and certified/reference values from SRM 2975 assigned by NIST and data from the literature

PAHs	This work, E1	This work, E3	NIST [33]	Schantz et al. [36]^c^	Schantz et al. [36]^d^	Sadiktsis et al. [34]^e^	Masala et al. [35]^e^
Phe	23.04 (0.43)	22.8 (0.4)	17.0 (2.8)^a^	20.8 (0.4)	20.510 (1.450)	23.7 (1.1)	ND
Ant	0.798 (0.037)	3.38 (0.19)	0.038 (0.008)^b^	0.0486 (0.0014)	0.0477 (0.0014)	0.661 (0.661)	ND
Flu	30.8 (0.3)	30.7 (1.0)	26.6 (5.1)^a^	31.2 (0.5)	31.0 (0.320)	30.2 (1.4)	ND
Pyr	2.03 (0.05)	1.86 (0.06)	0.90 (0.24)^a^	1.440 (0.05)	1.460 (0.040)	2.24 (0.13)	ND
B[a]A	1.80 (0.034)	1.502 (0.031)	0.317 (0.066)^a^	0.956 (0.044)	0.988 (0.056)	1.89 (0.09)	ND
Chr	9.75 (0.35)	9.23 (0.28)	4.56 (0.16)^a^	5.730 (0.05)	5.760 (0.280)	10.8 (0.6)	ND
B[b]F	18.4 (0.5)	20.4 (2.08)	11.5 (3.6)^b^	ND	ND	19.7 (2.1)	16.80 (1.77)
B[k]F	1.59 (0.09)	1.93 (0.19)	0.678 (0.076)^b^	1.750 (0.07)	1.790 (0.080)	1.56 (0.29)	1.61 (0.235)
B[a]P	0.803 (0.037)	0.657 (0.059)	0.052 (0.005)	0.773 (0.04)	0.770 (0.020)	0.919 (0.095)	0.870 (0.097)
I[1,2,3-cd]P	1.82 (0.09)	1.98 (0.12)	1.4 (0.2)^b^	2.120 (0.11)	2.60 (0.120)	1.52 (0.17)	1.97 (0.213)
DB[ah]A	0.218 (0.020)	0.250 (0.127)	0.52 (0.08)^a^	ND	ND	0.310 (0.053)	0.402 (0.043)
DB[al]P	0.079 (0.014)	0.026 (0.012)	ND	ND	ND	0.00611 (0.00350)	0.020 (0.001)
DB[ae]P	0.304 (0.057)	0.206 (0.026)	0.57^f^	0.616 (0.032)	0.606 (0.033)	0.292 (0.038)	0.226 (0.034)
DB[ai]P	0.056 (0.016)	0.049 (0.025)	ND	ND	ND	0.0386 (0.009)	0.035 (0.003)
DB[ah]P	0.019 (0.003)	0.025 (0.009)	ND	ND	ND	0.0275 (0.008)	0.017 (0.003)

*ND* no data reported

^a^Certified mass fraction for PAHs in SRM 2975

^b^Reference concentrations for PAHs in SRM 2975

^c^Method 16 (parameters for this PLE method are the same as those of E3)

^d^Method 18 (parameters for this PLE method are the same as those of E1

^e^200 °C with toluene/methanol used for PLE

^f^Information value

**Table 12:** Comparison of the determined PAHs from the present study by two-sided *t* tests with Bonferroni correction. The table lists significant differences for comparisons of extraction methods. Single (+/−) and double (++/−−) indicate statistically significant differences on the 5 % level without and with Bonferroni correction, respectively. The single plus or minus differences can be seen as an indication of a potential difference, while double plus or minus indicate stronger evidence for a true difference. No symbol indicates that no differences could found. ND indicates that no data was available and therefore the comparison could not be performed

1649a/b						1650b									2975										
PAHs	E1/E2 1649a, this study	E1/NIST 1649a [31]	E2/NIST 1649a [31]	E1/Schantz et al., 1649b [36]^a^	E2/Schantz et al., 1649b [36]^a^	E1/E3 1650 b, this study	E1/NIST [32]	E3/NIST [32]	E1/Schantz et al. [36]^b^	E3/Schantz et al. [36]^b^	E1/Schantz et al. [36]^c^	E3/Schantz et al. [36]^c^	E1/Sadiktsis et al. [34]^c^	E3/Sadiktsis et al. [34]^c^	E1/E3 2975, this study	E1/NIST [33]	E3/NIST [33]	E1/Schantz et al. [36]^b^	E3/Schantz et al. [36]^b^	E1/Schantz et al. [36]^c^	E3/Schantz et al. [36]^c^	E1/Sadiktsis et al. [34]^c^	E3/Sadiktsis et al. [34]^c^	E1/Masala et al. [35]^c^	E3/Masala et al. [35]^c^
Phe		+	+	+	+	−−		+	−	++	−	++	−	+		++	++	+	+	+				ND	ND
Ant		++	+	−		−−		++		++		++		++	−−	++	++	++	++	++	++	+	++	ND	ND
Flu						−	−−		−−	−			−											ND	ND
Pyr						−	−−		−−	−	−		−	+	+	++	++	++	++	++	++	−	−	ND	ND
B[a]A							++	++		−	−	−	+	+	++	++	++	+	++	+	++	−	−−	ND	ND
Chr		+	+	++	++	−	++	+	+	+	+	+	−			++	++	++	++	++	++	−	−	ND	ND
B[b]F				ND	ND	+	++		ND	ND	ND	ND		−			+	ND		ND					+
B[k]F	+		++		+	+	+		+		+			−−	−	++	++	−		−					
B[a]P					−	+	++	+		−		−	+		+	++	++		−				−		−
I[1,2,3-cd]P	+	−		−				−		−		−	+			+	+	−		−−	−	+	+		
DB[ah]A		++		ND	ND				ND	ND	ND	ND		−		−−	−−	ND	ND	ND	ND	−		−	−
DB[al]P				ND	ND	+	−	−	ND	ND	ND	ND	+	+	+	ND	ND	ND	ND	ND	ND	++	+	++	
DB[ae]P					−		−	−	−−	−−	−−	−−				ND	ND	−−	−−	−−	−−		−		
DB[ai]P				ND	ND		ND	ND	ND	ND	ND	ND				ND	ND	ND	ND	ND	ND				
DB[ah]P				ND	ND	−	ND	ND	ND	ND	ND	ND				ND	ND	ND	ND	ND	ND				

^a^Parameters in this PLE method are the same as those of E2

^b^Parameters in this PLE method are the same as those of E3

^c^Parameters are the same as those of E1

#### SRM 1649a

No significant difference was found in the PAH mass fraction when extracting SRM 1649a with E1 and E2 (Tables 9 and 12). Comparing the results from E1 and E2 to the NIST certified/reference values, significantly higher concentrations were obtained in the present study for Ant and DB[ah]A with E1 and for B[k]F using E2, respectively [31]. The significantly higher values determined for Ant and DB[ah]A could be a result of the extraction method employed in the present work as the data correspond better with the values reported in a recent relevant publication [36], where a higher extraction temperature was found to give increased concentrations of some PAHs such as Ant. Our results show also that the data for the other PAHs correspond well with the data reported by Schantz and co-workers [36], except for Chr where a significantly higher concentration was obtained (Tables 9 and 12) likely as a result of the chromatographic overlap of Chr with triphenylene. It should also be mentioned that the concentrations of PAHs in SRM 1649b are approximately 5 % lower than in SRM 1649a [43].

#### SRM 1650b

Tables 10 and 12 show the determined mass fractions for SRM 1650b using PLE methods E1 and E3. The PAHs Ant and Phe showed significantly lower mass fractions with E1 compared to E3, and a possible reason for this difference could be the use of methanol in the extraction solvent in E1. The extractions of this material with E1 and E3 show discrepancies in the results regarding the different PAHs compared to the values assigned by the NIST. However, a significant difference between this study and a study by Schantz and co-workers was only observed for DB[ae]P when similar extraction conditions were used, i.e., Tol:MeOH 9:1. No significant differences in the mass fractions of the PAHs (except for DB[al]P) were found using E1 compared to data from the study by Sadiktsis and co-workers [34], while significant differences were observed for Ant and B[a]A using E3 compared with the data from Sadiktsis et al.

#### SRM 2975

Tables 11 and 12 show the determined PAH mass fractions using E1 and E3 for SRM 2975. A lower mass fraction could be seen for Ant using E1 compared to E3, which was also observed for the other SRMs. Generally, the extraction methods used in this study gave higher mass fractions compared with the NIST certificate of analysis, except for Flu, Pyr, B[k]F, and DB[a,h]A. Interestingly, E1 and E3 gave either significantly higher or lower mass fractions for almost all of the PAHs when compared with the work by Schantz and co-workers [36] although similar extraction conditions were used. No significant difference can be observed between E1 and previously published results from our research group using the same extraction conditions as E1, except for DB[al]P. One of the reasons for the discrepancies between this study and the study by Schantz and co-workers [36] could be due to inhomogeneous particle distribution in different bottles of SRM 2975, which the NIST has stated in the certificate of analysis for this material [33].

## Conclusions

An analytical setup comprising PLE, SPE, and online LC–GC/MS for the analysis of OPAHs has been evaluated. The determined LODs for the analyzed OPAHs are generally lower than those previously reported in the literature. The utilization of LVI enables low LODs, which is important in the analysis of OPAHs in low amounts of PM. Furthermore, coupling parts of the analytical protocol online reduces manual labor. By evaluating three different PLE methods for three separate SRMs, it can be concluded that the tested methods were not optimal for the simultaneous analysis of all PAHs and OPAHs in diesel particles. However, good agreement was found for the analysis of PAHs and OPAHs in urban air particles using the two different PLE methods. The variation in the results from this study and data from literature show the importance of the assessment of certified values of OPAHs in these SRMs.

## Electronic supplementary material

Below is the link to the electronic supplementary material.

ESM 1 (PDF 49 kb)

## References

[1] Sehlstedt M, Forsberg B, Westerholm R, Boman C, Sandström T (2007) http://www20.vv.se/fudresultat/Publikationer_000301_000400/Publikation_000310/EMFO20litteraturstudie%20Trafikrelaterade%20partiklar%20och%20h%C3%A4lsoeffekter%20-%20Final%20report%20071212.pdf. Accessed July 2014

[2] Kunzli N, Kaiser R, Medina S, Studnicka M, Chanel O, Filliger P, Herry M, Horak FJ, Puybonnieux-Texier V, Quenel P, Schneider J, Seethaler R, Vergnaud JC, Sommer H (2000). Lancet.

[3] Brunekreef B, Holgate ST (2002). Lancet.

[4] See SW, Balasubramanian R, Yang TS, Karthikeyan S (2006). J Toxic Environ Health A.

[5] European Commission (2005) The communication from the commission to the council and the European Parliament on thematic strategy on air pollution and the directive on ambient air quality and cleaner air for Europe, impact assessment. Brussels, September 2005. http://ec.europa.eu/environment/archives/cafe/pdf/ia_report_en050921_final.pdf. Accessed July 2014

[6] Mauderly JL (2001). Toxicol Sci.

[7] US Environmental Protection Agency (2002) Health assessment document for diesel engine exhaust, EPA/600/8-90/057F. http://www.epa.gov/ttn/atw/dieselfinal.pdf. Accessed July 2014

[8] Singh P, DeMarini DM, Dick CAJ, Tabor DG, Ryan JV, Linak WP (2003). Environ Health Perspect.

[9] IRAC (2010). Monographs on the evaluation of carcinogenic risks to humans: carbon black, titanium dioxide and talc.

[10] Lewtas J (2007). Mutat Res.

[11] Lundstedt S, White PA, Lemieux CL, Lynes KD, Lambert IB, Oberg L, Haglund P, Tysklind M (2007). Ambio.

[12] Wang W, Jariyasopit N, Schrlau J, Jia Y, Tao S, Yu TW, Dashwood RH, Zhang W, Wang X, Simonich SL (2011). Environ Sci Technol.

[13] Goldfarb JL, Suuberg EM (2008). Environ Toxicol Chem.

[14] Vione D, Barra S, De Gennaro G, De Rienzo M, Gilardoni S, Perrone MG, Pozzoli L (2004). Ann Chim.

[15] Eiguren-Fernandez A, Miguel AH, Di Stefano E, Schmitz DA, Cho Ar K, Thurairatnam S, Avol EL, Froines JR (2008). Aerosol Sci Tech.

[16] Alsberg T, Stenberg U, Westerholm R, Strandell M, Rannug U, Sundvall A, Romert L, Bernson V, Pettersson B, Toftgtird R, Franzqn B, Jansson M, Gustafsson JK, Egebiick KE, Tejle G (1985). Environ Sci Technol.

[17] Jakober CA, Riddle SG, Robert MA, Destaillats H, Charles MJ, Green PG, Kleeman MJ (2007). Environ Sci Technol.

[18] Walgraeve C, Demeestere K, Dewulf J, Zimmermann R, Van Langenhove H (2010). Atmos Environ.

[19] Harrad SHS, Callén Romero MS, Harrison RM (2003). Atmos Environ.

[20] Lintelmann J, Fischer K, Matuschek G (2006). J Chromatogr A.

[21] Delhomme O, Millet M, Herckes P (2008). Talanta.

[22] Castells P, Santos FJ, Galceran MT (2003). J Chromatogr A.

[23] Albinet A, Leoz-Garziandia E, Budzinski H, Viilenave E (2006). J Chromatogr A.

[24] Mirivel G, Riffault V, Galloo JC (2010). Anal Bioanal Chem.

[25] Layshock JA, Wilson G, Anderson KA (2010). Environ Toxicol Chem.

[26] Nocun MS, Schantz MM (2013). Anal Bioanal Chem.

[27] Kuosmanen K, Hyötyläinen T, Hartonen K, Jönsson JA, Riekkola ML (2003). Anal Bioanal Chem.

[28] Christensen A, Ostman C, Westerholm R (2005). Anal Bioanal Chem.

[29] Bergvall C, Westerholm R (2006). Anal Bioanal Chem.

[30] Warner SD, Farant JP, Butler IS (2004). Chemosphere.

[31] NIST (2007). Certificate of analysis. SRM 1649a. Urban dust.

[32] NIST (2006). Certificate of analysis. SRM1650b. Diesel particulate matter.

[33] NIST (2009). Certificate of analysis. SRM 2975. Diesel particulate matter (industrial forklift).

[34] Sadiktsis I, Koegler JH, Benham T, Bergvall C, Westerholm R (2014). Fuel.

[35] Masala S, Ahmed T, Bergvall C, Westerholm R (2011). Anal Bioanal Chem.

[36] Schantz MM, McGaw E, Wise SA (2012). Anal Chem.

[37] Walgraeve C, Demeestere K, De Wispelaere P, Dewulf J, Lintelmann J, Fischer K, Van Langenhove H (2012). Anal Bioanal Chem.

[38] Lintelmann J, Fischer K, Karg E, Schroppel A (2005). Anal Bioanal Chem.

[39] O’Connell SG, Haigh T, Wilson G, Anderson KA (2013). Anal Bioanal Chem.

[40] Cochran RE, Dongari N, Jeong H, Beranek J, Haddadi S, Shipp J, Kubatova A (2012). Anal Chim Acta.

[41] European Commission (2005) http://www.fsai.ie/uploadedFiles/Commission_Recommendation_2005_108_EC.pdf. Accessed July 2014

[42] US Environmental Protection Agency (2008) Polycyclic aromatic hydrocarbons http://www.epa.gov/osw/hazard/wastemin/minimize/factshts/pahs.pdf. Accessed July 2014

[43] NIST (2009). Certificate of analysis, SRM 1649b.

[44] Cho AK, Stefano E, You Y, Rodriguez CE, Schmitz DA, Kumagai Y, Miguel AH, Eiguren-Fernandez A, Kobayashi T, Avol E, Froines JR (2004). Aerosol Sci Technol.

[45] Fernandez P, Bayona J (1992). J Chromatogr.

[46] Durant J, Lafleuran A, Plummer E, Taghizadeh K, Busby W, Thilly W (1998). Environ Sci Technol.

[47] Sun Q, Alexandrova OA, Herckes P, Allen JO (2009). Talanta.

