# Supplementary material for: Determination of oxygenated and native polycyclic aromatic hydrocarbons in urban dust and diesel particulate matter standard reference materials using pressurized liquid extraction and LC–GC/MS
Source: Anal Bioanal Chem. 2014 Nov 14;407(2):427–38. doi: 10.1007/s00216-014-8304-8 (PMC4300434; doi:10.1007/s00216-014-8304-8)
Supplement: Supplementary file 1 — (PDF 49 kb) [file 216_2014_8304_MOESM1_ESM.pdf]

Analytical and Bioanalytical Chemistry

Electronic Supplementary Material

Determination of oxygenated and native polycyclic aromatic hydrocarbons in urban dust and diesel particulate matter standard reference materials using pressurized liquid extraction and LC-GC/MS

Trifa M. Ahmed, Christoffer Bergvall , Magnus Åberg, Roger Westerholm

Table S1 List of PAH standards and surrogate internal standards with abbreviation, supplier and purity

Name	Abbreviation	Supplier	Purity (%)
Phenanthrene-D_{10}	Phe-D_{10}	Chiron AS, Trondheim, Norway[a)], Larodan Fine Chemicals AB, Sweden[b)]	99.3[a)], 97.2[b)]
Phenanthrene	Phe	Merck, Germany	98.7
Anthracene	Ant	Sigma-Aldrich, St. Louis, MO, USA	99.6
3-Methylphenanthrene	3-MPhe	Larodan Fine Chemicals AB, Sweden	99.9
2-Methylphenanthrene	2-MPhe	Sigma-Aldrich, St. Louis, MO, USA	93.8
2-Methylanthracene	2-MAnt	Koch-Light Laboratories, UK	100
9-Methylphenanthrene	9-MPhe	Chiron AS, Trondheim, Norway	100
1-Methylphenanthrene	1-MPhe	Larodan Fine Chemicals AB, Sweden	98.8
4*H*-Cyclopenta[*def*]phenanthrene	4H-CPP	Sigma-Aldrich, St. Louis, MO, USA	99.1
3,6-Dimethylphenanthrene	3,6-DMPhe	Larodan Fine Chemicals AB, Sweden	96.1
2-Phenylnaphthalene	2-PhNap	EGA-Chemie, Steinheim, Germany	94.2
9-Methylanthracene	9-MAnt	Koch-Light Laboratories, UK	97.1
3,9-Dimethylphenanthrene	3,9-DMPhe	Chiron AS, Trondheim, Norway	99.7
Fluoranthene	Flu	Sigma-Aldrich, St. Louis, MO, USA	97.2
Pyrene-D_{10}	Pyr-D_{10}	Chiron AS, Trondheim, Norway[a)], Larodan Fine Chemicals AB, Sweden[b)]	99.8[a)], 95.7[b)]
Pyrene	Pyr	Janssen Chimica, Belgium	97.6
1-Methylfluoranthene	1-MFlu	Chiron AS, Trondheim, Norway	99.7

Benzo[*a*]fluorene	B[a]f	Chiron AS, Trondheim, Norway	98.8
Benzo[*b*]fluorene	B[b]f	Sigma-Aldrich, St. Louis, MO, USA	99.1
2-Methylpyrene	2-MPyr	Chiron AS, Trondheim, Norway	98.3
4-Methylpyrene	4-MPyr	Chiron AS, Trondheim, Norway	99.6
1-Methylpyrene	1-MPyr	Larodan Fine Chemicals AB, Sweden	99.1
Benzo[*c*]phenanthrene	B[c]Phe	Chiron AS, Trondheim, Norway	99.5
Benzo[*ghi*]fluoranthene	B[ghi]F	Larodan Fine Chemicals AB, Sweden	99.5
Benzo[*b*]naphto[1,2-*d*]thiophene	B[b]NT	Chiron AS, Trondheim, Norway	99.6
Benz[*a*]anthracene-D_{12}	B[a]A-D_{12}	Chiron AS, Trondheim, Norway	98.6
Benz[*a*]anthracene	B[a]A	Fluka AG, Switzerland	98.4
Chrysene	Chr	Sigma-Aldrich, St. Louis, MO, USA	96.4
3-Methylchrysene	3-MChr	Chiron AS, Trondheim, Norway	99.1
2-Methylchrysene	2-MChr	Chiron AS, Trondheim, Norway	99.7
6-Methylchrysene	6-MChr	Chiron AS, Trondheim, Norway	100
1-Methylchrysene	1-MChr	Chiron AS, Trondheim, Norway	99.3
Benzo[*b*]fluoranthene	B[b]F	Chem Service, West Chester, PA, USA	100
Benzo[*k*]fluoranthene	B[k]F	Chem Service, West Chester, PA, USA	98.3
Benzo[*e*]pyrene	B[e]P	Sigma-Aldrich, St. Louis, MO, USA	99.7
Benzo[*a*]pyrene-D_{12}	B[a]P-D_{12}	Chiron AS, Trondheim, Norway[a)], Larodan Fine Chemicals AB, Sweden[b)]	98.7[a)], 98.1[b)]

Benzo[*a*]pyrene	B[a]P	Sigma-Aldrich, St. Louis, MO, USA	97.6
Perylene	Per	Sigma-Aldrich, St. Louis, MO, USA	99.5
Indeno[1,2,3-*cd*]fluoranthene	I[1,2,3-cd]F	Radiant Dyes, Wermelskirchen, Germany	98.4
Indeno[1,2,3-*cd*]pyrene	I[1,2,3-cd]P	AccuStandard Inc., New Haven, CT, USA	99.8
Dibenz[*a,h*]anthracene	DB[a,h]A	Fluka AG, Switzerland	99.4
Picene	Pic	Larodan Fine Chemicals AB, Sweden	96.1
Benzo[*ghi*]perylene-D_{12}	B[ghi]P-D_{12}	Chiron AS, Trondheim, Norway	99.6
Benzo[*ghi*]perylene	B[ghi]p	Janssen Chimica, Belgium	98.8
Dibenzo[a,l]pyrene	DB[a,l]P	AccuStandard Inc., New Haven, CT, USA	96
Dibenzo[a,e]pyrene	DB[a,e]	LGC Promochem, Sweden	98
Coronene-D_{12}	Cor-D_{12}	Chiron AS, Trondheim, Norway	98.9
Coronen	Cor	Radiant Dyes. Wermelskirchen, Germany	100
Dibenzo[a,i]pyrene-D_{14}	DB[a,i]P-D_{14}	LGC Promochem, Sweden	98
Dibenzo[a,i]pyrene	DB[a,i]P	Sigma-Aldrich, St. Louis, MO, USA	96.4
Dibenzo[*a,h*]pyrene	DB[a,h]P	Koch-Light Laboratories, UK	100

Table S2 Mass fractions in ng mg^{-1} for SRM 1649a (n= 3, with standard deviations) using E1 and E2 methods, certified/reference values for SRM 1649a assigned by NIST and data for SRM 1649b by Schantz

PAHs	Abbreviations	This work E1	This work E2	NIST [31]	Schantz et al.[36][c]
3-Methylphenanthrene	3-MPhe	0.830 (0.046)	0.904 (0.214)	0.50 (0.05)[b]	0.640 (0.045)
2-Methylphenanthrene	2-MPhe	1.21 (0.06)	1.34 (0.30)	0.73 (0.12)[b]	0.920 (0.073)
2-Methylanthracene	2-MAnt	0.193 (0.017)	0.237 (0.067)	N/D	N/D
9-Methylphenanthrene	9-MPhe	0.518 (0.20)	0.601 (0.154)	N/D	0.385 (0.034)
1-Methylphenanthrene	1-MPhe	0.566 (0.027)	0.687 (0.170)	0.37 (0.04)[b]	0.549 (0.028)
4H-cyclopenta[def]phenanthrene	4H-CPP	0.286 (0.052)	0.262 (0.011)	0.32 (0.06)	N/D
9-Methylanthracene	9-MAnt	0.080 (0.006)	0.109 (0.028)	N/D	N/D
2-Phenylnaphthalene	2-PhNap	0.494 (0.059)	0.423 (0.028)	N/D	N/D
3.6-Dimethylphenanthrene	3.6-DMPhe	0.117 (0.005)	0.107 (0.002)	N/D	N/D
3.9-Dimethylphenanthrene	3.9-DMPhe	0.439(0.034)	0.423 (0.016)	N/D	N/D
1-Methylfluoranthene	1-MFlu	0.537 (0.049)	0.561 (0.063)	N/D	0.115 (0.009)
Benz[a]fluorene	B[a]F	0.390 (0.048)	0.366 (0.031)	N/D	N/D
Benz[b]fluorene	B[b]F	0.153 (0.021)	0.133 (0.017)	N/D	N/D
2-Methylpyrene	2-MPyr	0.405 (0.030)	0.438 (0.039)	N/D	N/D
4-Methylpyrene	4-MPyr	0.367 (0.020)	0.368 (0.040)	N/D	N/D
1-Methylpyrene	1-MPyr	0.278 (0.016)	0.290 (0.029)	N/D	0.343 (0.027
Benzo[ghi]fluoranthene	B[ghi]F	1.10 (0.02)	1.00 (0.034)	0.88 (0.02)[b]	0.954 (0.044)
Benzo[c]phenanthrene	B[c]PHE	0.460 (0.031)	0.466 (0.018)	0.46 (0.03)[b]	0.433 (0.019)
Benzo[b]naphto[1,2-d]thiophene	B[b]NAP	0.145 (0.003)	0.145 (0.014)	N/D	N/D
3-Methylchrysene	3-MChr	0.304 (0.038)	0.335 (0.016)	N/D	0.279 (0.014)
2-Methylchrysene	2-MChr	0.603 (0.050)	0.539 (0.018)	N/D	N/D
6-Methylchrysene	6-MChr	0.239 (0.012)	0.239(0.019)	N/D	N/D
1-Methylchrysene	1-MChr	0.283 (0.017)	0.273(0.020)	N/D	N/D
Benzo[e]pyrene	B[e]P	3.36 (0.11)	3.52 (0.18)	3.090 (0.19)[a]	2.998 (0.099)
Perylene	Per	0.691 (0.035)	0.656 (0.059)	0.646 (0.075)[a]	0.624 (0.028)
Indeno[1, 2,3-cd]fluoranthene	I[1,2,3-cd]F	0.269 (0.017)	0.227 (0.008)	N/D	ND
Picene	Pic	0.515 (0.045)	0.439 (0.004)	0.426 (0.022)[a]	0.380 (0.021)
Benzo[ghi]perylene	B[ghi]P	4.16 (0.10)	3.87 (0.07)	4.010 (0.91)[a]	4.220 (0.041)
Coronene	Cor	6.55 (0.09)	4.52 (0.63)	N/D	3.290 (0.180)

N/D=no data reported, a=Certified mass fraction for PAHs in SRM 1649a, b=Reference mass fraction for PAHs in SRM 1649a, c=method 8(200°C and toluene used as extraction solvent)

Table S3 Mass fractions in ng mg^{-1} for SRM 1650b (n= 3, with standard deviations) for the PAHs extracted from 1650b using E1 and E3 methods and certified/reference values assigned by NIST and data from the literature

PAHs	This work, E1	This work, E3	NIST [32]	Schantz et al., [36][c]	Schantz et al., [36][d]	Sadiktsis et al.,[34][e]
3-MPhe	52.8 (6.7)	73.9 (8.1)	55.1 (1.9)[a]	58.8 (4.4)	ND	75.5 (4.4)
2-MPhe	74.5 (9.5)	98.9 (10.0)	70.7 (2.7)[a]	71.2 (5.6)	ND	96.4 (4.3)
2-MAnt	3.84 (1.06)	12.7 (1.6)	5.88 (0.32)[a]	ND	ND	4.60 (0.30)
9-MPhe	33.7 (4.9)	47.7 (5.1)	35.1 (1.9)[a]	38.5 (9)	ND	47.4 (3.3)
1-MPhe	30.1 (4.6)	42.0 (1.8)	28.3 (1.5)[a]	32.3 (1.8)	ND	43.0 (2.6)
4H-CPP	1.52 (0.28)	2.79 (0.64)	3.34 (0.16)[b]	ND	ND	1.96 (0.2)
9-MAnt	3.28 (0.81)	7.83 (1.94)	N/D	ND	ND	47.4 (3.3)
2-PhNAP	6.69 (0.84)	5.21 (0.92)	N/D	ND	ND	6.32 (0.25)
3,6-DMPhe	11.3 (1.5)	10.1 (1.5)	23 (2)[b]	ND	ND	11.5 (0.5)
3,9-DMPhe	38.1 (0.9)	34.6 (5.2)	N/D	ND	ND	36.6 (1.5)
1-MFlu	9.71 (0.71)	10.9 (0.9)	3.09 (0.07)[b]	ND	ND	9.61 (0.66)
B[a]f	1.45 (0.10)	1.6 (0.2)	N/D	ND	ND	1.32 (0.09)
B[b]f	0.540 (0.055)	0.388 (0.024)	N/D	ND	ND	0.337 (0.021)
2-MPyr	4.56 (3.61)	6.11 (0.35)	5.8 (1.2)[b]	ND	ND	5.87 (0.31)
4-MPyr	5.28 (0.40)	5.50 (0.92)	5.14 (0.57)[b]	ND	ND	5.22 (0.28)
1-MPyr	1.84 (0.20)	2.06 (0.82)	2.06 (0.27)[b]	ND	ND	1.70 (0.09)
B[ghi]F	12.4 (0.5)	12.0 (0.4)	10.8 (1.0)[a]	11.80 (0.60)	11.280 (1.010)	12.0 (0.1)
B[c]Phe	3.05 (0.20)	3.04 (1.80)	2.51 (0.29)[a]	2.95 (0.26)	2.890 (0.200)	3.19 (0.04)
B[b]NT	1.30 (0.02)	1.25 (0.50)	N/D	ND	ND	1.13 (0.03)
3-MChr	2.37 (0.08)	1.97 (0.49)	2.10 (0.13)[b]	2.30 (0.20)	ND	1.55 (0.02)
2-MChr	3.92 (0.13)	3.05 (0.07)	2.50 (0.20)[b]	ND	ND	3.09 (0.02)
6-MChr	1.75 (0.07)	1.58 (0.11)	1.58 (0.03)[b]	1.53 (0.15)	ND	1.55 (0.03)
1-MChr	2.15 (0.03)	1.75 (0.56)	1.47 (0.04)[b]	ND	ND	1.68 (0.08)
B[e]P	7.40 (0.57)	6.88 (0.10)	6.30 (0.5)[a]	6.57 (0.51)	6.500 (0.080)	7.13 (0.16)
Per	0.174 (0.006)	0.169 (0.049)	0.165 (0.032)	0.169 (0.01)	0.158 (0.012)	0.120 (0.007)
I[1,2,3-cd]F	0.288 (0.034)	0.181 (0.025)	N/D	ND	ND	0.197 (0.024)
Pic	0.679 (0.034)	0.444 (0.044)	0.499 (0.061)[a]	0.52 (0.04)	0.519 (0.024)	0.593 (0.056)
B[ghi]P	6.78 (0.36)	5.73 (0.12)	5.91 (0.18)[a]	7.01 (0.12)	7.040 (0.220)	6.01(0.04)
Cor	6.09 (0.62)	6.42 (3.09)	9.6 (1.0)[b]	9.76 (0.32)	9.810 (0.250)	5.02 (0.66)

N/D=no data reported, a=Certified mass fraction for PAHs in SRM 1650b, b=Reference mass fraction for PAHs in SRM 1650b, c=method 12(200°C with toluene used for PLE), d=method 17(200°C with toluene/methanol used for PLE), e=200°C with toluene/methanol used for PLE

Table S4 Mass fractions in ng mg^{-1} for SRM 2975 (n= 3, with standard deviations) for the PAHs extracted from 1650b using E1 and E3 methods and certified/reference values assigned by NIST and data from the literature

PAHs	This work, E1	This work, E3	NIST, [33]	Schantzet al., [36][d]	Schantz et al.,[36][e]	Sadiktsis et al., [34][f]	Masala et al.,[35][f]
3-MPhe	1.14 (0.03)	1.17 (0.07)	1.0 (0.2)[b]	1.320 (0.130)	ND	0.306 (0.023)	N/D
2-MPhe	2.62 (0.04)	2.80 (0.14)	2 (0.2)[b]	2.60 (0.19)	ND	ND	N/D
2-MAnt	0.104 (0.015)	0.482 (0.064)	N/D	ND	ND	ND	N/D
9-MPhe	0.398 (0.005)	0.445 (0.030)	0.44 (0.09)[c]	0.570 (0.03)	ND	0.0945 (0.0100)	N/D
1-MPhe	0.986 (0.014)	1.16 (0.07)	0.89 (0.11)	0.967 (0.064)	ND	0.240 (0.027)	N/D
4H-CPP	0.034 (0.003)	0.039 (0.003)	N/D	ND	ND	0.00985 (0.0004)	N/D
9-MAnt	0.016 (0.006)	0.021 (0.001)	N/D	ND	ND	0.00208 (0.00051)	N/D
2-PhNap	3.17 (0.23)	2.78 (0.14)	N/D	ND	ND	2.92 (0.40)	N/D
3,6-DMPhe	0.064 (0.004)	0.065 (0.004)	0.18 (0.02)[b]	ND	ND	0.0511 (0.0039)	N/D
3,9-DMPhe	0.148 (0.001)	0.190 (0.016)	N/D	ND	ND	0.12 (0.020)	N/D
1-MFlu	0.439 (0.118)	0.401 (0.036)	0.53 (0.03)	ND	ND	0.530 (0.072)	N/D
B[a]f	0.464 (0.356)	0.290 (0.198)	N/D	ND	ND	0.0303 (0.0064)	N/D
B[b]f	0.489 (0.375)	0.037 (0.018)	N/D	ND	ND	0.0256 (0.0043)	N/D
2-MPyr	0.071 (0.002)	0.090 (0.004)	0.040 (0.008)[b]	ND	ND	0.0668 (0.0096)	N/D
4-MPyr	0.042 (0.001)	0.033 (0.002)	0.022 (0.005)[b]	ND	ND	0.0341(0.0035)	N/D
1-MPyr	0.042 (0.009)	0.110 (0.005)	N/D	ND	ND	0.0313 (0.0054)	N/D
B[ghi]F	12.5 (0.4)	13.5 (0.5)	10.2 (0.5)[b]	10.9 (0.3)	10.670 (0.900)	15.4 (0.8)	N/D
B[c]Phe	1.44 (0.06)	1.39 (0.08)	1.0 (0.4)[b]	1.66 (0.12)	1.620 (0.110)	1.58 (0.10)	N/D
B[b]NT	0.093 (0.004)	0.109 (0.007)	N/D	ND		0.106 (0.007)	N/D
3-MChr	0.053 (0.002)	0.044 (0.002)	N/D	ND	ND	0.0345 (0.0022)	ND
2-MChr	0.094 (0.001)	0.005 (0.002)	N/D	ND	ND	0.0986 (0.0049)	ND
6-MChr	0.080 (0.049)	0.044 (0.042)	N/D	ND	ND	0.0236 (0.0009)	ND
1-MChr	0.046 (0.002)	0.045 (0.003)	N/D	ND	ND	0.0439 (0.0015)	ND
B[e]P	2.30 (0.06)	2.53 (0.22)	1.11 (0.1)[a]	2.370 (0.12)	2.380 (0.040)	2.57 (0.23)	2.26 (0.243)
Per	0.073 (0.003)	0.066 (0.004)	0.054 (0.009)[b]	0.0845 (0.0065)	0.0837 (0.0052)	0.0838 (0.0077)	0.074 (0.009)
IcdF	0.794 (0.056)	0.782 (0.059)	1.1 (0.2)[b]	ND	ND	0.664 (0.065)	0.853 (0.097)
Pic	0.796 (0.053)	0.817 (0.044)	1.0 (0.2)[b]	0.926 (0.019)	0.929 (0.015)	0.653 (0.090)	0.976 (0.92)
B[ghi]P	1.30 (0.03)	1.19(0.10)	0.498 (0.044)[a]	1.570 (0.14)	1.560 (0.070)	1.25 (0.05)	1.31 (0.285)
Cor	4.68 (0.25)	2.74 (0.37)	1.1 (0.2)[b]	2.050 (0.16)	2.120 (0.120)	5.94 (0.52)	5.07 (0.414)

N/D =no data reported, a=Certified mass fraction for PAHs in SRM 2975, b=Reference mass fraction for PAHs in SRM 2975, c=sum of 4- and 9-Methylphenanthrene, d=mehtod16 (Toluene used as extraction solvent with 200°C), e= method 18(Toluene/Methanol used as extraction solvent with 200°C), f=200°C with toluene/methanol used for PLE

Table S5 Comparison of the determined PAHs from the present study by two-sided t-tests with Bonferroni correction. The table lists significant differences for comparisons of extraction methods. Single (+/-) and double (++/--) indicate statistically significant differences on the 5 % level without and with Bonferroni correction, respectively. The single plus or minus differences can be seen as an indication of a potential difference, while double plus or minus indicate stronger evidence for a true difference. No symbol indicates that no differences could found. *N.D.* indicates that no data was available and therefore the comparison could not be performed

SRM 1649a/b						SRM 1650b									SRM 2975										
PAHs	E1/ E2 1649a this study	E1/ NIST, [31]	E2 / NIST, [31]	E1 / Schantz et al., 1649b[26][a]	E2 / Schantz et al., 1649b[26][a]	E1 / E3 1650 b this study	E1/ NIST, [32]	E3 / NIST, [32]	E1/ Schantz et al., [26][b]	E3 / Schantz et al., [26][b]	E1/ Schantz et al., [26][c]	E3 / Schantz et al., [26][c]	E1 / Sadiktsis et al., [34][c]	E3 / Sadiktsis et al., [34][c]	E1/ E3 2975 this study	E1/ NIST, [33]	E3 / NIST, [33]	E1/ Schantz et al., [26][b]	E3 / Schantz et al[b]., [26][b]	E1 / Schantz et al., [26][c]	E3 / Schantz et al .,[26][c]	E1 / Sadiktsis et al., [34][c]	E3 / Sadiktsis et al., [34][c]	E1 / Masala et al., [35][c]	E3 / Masala et al., [35][c]
3-MPhe		++	+	+		-		++		+	ND	ND	-							ND	ND	++	++	ND	ND
2-MPhe		++	+	+		-		++		+	ND	ND	-			++	++			ND	ND	ND	ND	ND	ND
2-MAnt		ND	ND	ND	ND	--		+	ND	ND	ND	ND		++	--	ND	ND	ND	ND	ND	ND	ND	ND	ND	ND
9-MPhe	--	ND	ND	+	--	-		+		+	ND	ND	-			ND	ND	--	-	ND	ND	++	++	ND	ND
1-MPhe		++				-		+		+	ND	ND	-		-		--			ND	ND	++	++	ND	ND
4H-CPdefP			-	ND	ND	-	--		ND	ND	ND	ND				ND	ND	ND	ND	ND	ND	++	++	ND	ND
9-MANDt		ND	ND	ND	ND	-	ND	ND	ND	ND	ND	ND	-	+		ND	ND	ND	ND	ND	ND	+	++	ND	ND
2-PhNDap		ND	ND	ND	ND		ND	ND	ND	ND	ND	ND				ND	ND	ND	ND	ND	ND			ND	ND
3,6-DMPhe	-	ND	ND	ND	ND		ND	ND	ND	ND	ND	ND				--	--	ND	ND	ND	ND	+	+	ND	ND
3,9-DMPhe		ND	ND	ND	ND		ND	ND	ND	ND	ND	ND			-	ND	ND	ND	ND	ND	ND		+	ND	ND
1-MFlu		ND	ND	-			++	++	ND	ND	ND	ND				ND	ND	ND	ND	ND	ND		-	ND	ND
B[a]F		ND	ND	ND	ND		ND	ND	ND	ND	ND	ND		+		ND	ND	ND	ND	ND	ND		+	ND	ND
B[b]F		ND	ND	ND	ND	+	ND	ND	ND	ND	ND	ND	++	+		ND	ND	ND	ND	ND	ND			ND	ND
2-MPyr		ND	ND	ND	ND				ND	ND	ND	ND			-	+	+	ND	ND	ND	ND		+	ND	ND
4-MPyr		ND	ND	ND	ND				ND	ND	ND	ND			+	+		ND	ND	ND	ND	+		ND	ND
1-MPyr		ND	ND	ND	ND				ND	ND	ND	ND			--	ND	ND	ND	ND	ND	ND		++	ND	ND
B[ghi]F	-	++	+	+			+		ND	ND						++	++	+	++	+	+	-	-	ND	ND
B[c]PHE							++		ND	ND						+	+	-	-		-	-	-	ND	ND
B[b]NDAP		ND	ND	ND	ND		ND	ND	ND	ND	ND	ND	++		-	ND	ND	ND	ND	ND	ND	-		ND	ND

3-MChr		ND	ND	ND	ND		+		ND	ND	ND	ND	++	++	++	ND	ND	ND	ND	ND	ND	++	+	ND	ND
2-MChr		ND	ND	ND	ND	++	++	+	ND	ND	ND	ND	++			ND	ND	ND	ND	ND	ND	-	-	ND	ND
6-MChr		ND	ND	ND	ND		+		ND	ND	ND	ND	+			ND	ND	ND	ND	ND	ND			ND	ND
1-MChr		ND	ND	ND	ND	++	++	+	ND	ND	ND	ND	++			ND	ND	ND	ND	ND	ND			ND	ND
B[e]P		+	+	+	+		++					+				++	++								
Per													++						ND		ND		-		
I[1,2,3-cd]F	-	ND	ND	ND	ND	+	ND	ND	ND	ND	ND	ND	+			--	-	ND	ND	ND	ND	+			
Pic	-	+		+	+	+	+		+		+			-		-	-	--	-	--	-		+	-	-
B[ghi]P	-	+	-		--	+	++			--		--	+			++	++	-	-	-	-	+			
Cor	-	++		++	+		-	-	--	--	--	--		+	+	++	++	++	+	++	+	-	--		--

a=parameters in this PLE method are same as E2, b= parameters in this PLE method are same as E3, c= parameters are the same as E1
